# Real-time assessment of neutrophil metabolism and oxidative burst using extracellular flux analysis

**DOI:** 10.3389/fimmu.2023.1083072

**Published:** 2023-04-25

**Authors:** Frances S. Grudzinska, Alice Jasper, Elizabeth Sapey, David R. Thickett, Claudio Mauro, Aaron Scott, Jonathan Barlow

**Affiliations:** ^1^ Birmingham Acute Care Research Group, Institute of Inflammation and Ageing, University of Birmingham, Birmingham, United Kingdom; ^2^ PIONEER Health Data Research- UK Hub in Acute Care, Institute of Inflammation and Ageing, University of Birmingham, Birmingham, United Kingdom; ^3^ Cellular Health and Metabolism Facility, School of Sport, Exercise and Rehabilitation Sciences, University of Birmingham, Birmingham, United Kingdom

**Keywords:** neutrophils, extracellular flux analysis, immunometabolism, glycolysis, oxidative burst

## Abstract

Neutrophil responses are critical during inflammatory and infective events, and neutrophil dysregulation has been associated with poor patient outcomes. Immunometabolism is a rapidly growing field that has provided insights into cellular functions in health and disease. Neutrophils are highly glycolytic when activated, with inhibition of glycolysis associated with functional deficits. There is currently very limited data available assessing metabolism in neutrophils. Extracellular flux (XF) analysis assesses real time oxygen consumption and the rate of proton efflux in cells. This technology allows for the automated addition of inhibitors and stimulants to visualise the effect on metabolism. We describe optimised protocols for an XFe96 XF Analyser to (i) probe glycolysis in neutrophils under basal and stimulated conditions, (ii) probe phorbol 12-myristate 13-acetate induced oxidative burst, and (iii) highlight challenges of using XF technology to examine mitochondrial function in neutrophils. We provide an overview of how to analyze XF data and identify pitfalls of probing neutrophil metabolism with XF analysis. In summary we describe robust methods for assessing glycolysis and oxidative burst in human neutrophils and discuss the challenges around using this technique to assess mitochondrial respiration. XF technology is a powerful platform with a user-friendly interface and data analysis templates, however we suggest caution when assessing neutrophil mitochondrial respiration.

## Introduction

Neutrophils are vital in the host response to infection and inflammation. Neutrophil dysfunction is described at the extremes of age, in overwhelming infection, in chronic diseases and is associated with poor outcomes for patients (1, 2). In sepsis and other disease states there is broad dysfunction with all neutrophil functions reduced (3, 4). Ineffective neutrophil responses are harmful during the acute response where dysfunction may drive increased mortality and morbidity (5, 6). Then in the resolution phase persistent neutrophilic responses potentiates tissue damage as seen in acute respiratory distress syndrome (ARDS) (7) and unopposed neutrophil action in alpha-1 anti-trypsin deficiency (8). In patients with ARDS or sepsis, studies of neutrophil extracellular traps (NETs) demonstrate that there is an optimal level of *ex-vivo* function with poor outcomes for those with the highest or lowest levels of NETosis (6, 9). While alterations to a single cell function suggests involvement of a single molecular pathway, broad cellular dysfunction implicates a central driver such as altered metabolism. Immunometabolism is defined as the role of cellular metabolism in the regulation of immune cell function (10).

Murine work by Sadiku and colleagues has demonstrated that regulation of neutrophil function is linked to metabolism (11). Neutrophils are highly glycolytic (12) and inhibition of glycolysis using 2-deoxyglucose (2-DG) significantly reduces migration, phagocytosis, NETosis and oxidative burst. Neutrophils possess a functional network of mitochondria; however, mitochondrial oxidative phosphorylation does not appear to be required for adenosine triphosphate (ATP) synthesis to meet the energetic demand of neutrophils (13–15). The bulk of ATP is synthesised by glycolysis (16, 17). Sadiku et al. demonstrated that neutrophils from patients with chronic obstructive pulmonary disease had lower baseline ATP levels and exhibited reduced glycolysis in response to a pathogen compared to age matched controls (18). Moreover, the glycolytic capacity of neutrophils can be impacted by treatment with lipopolysaccharide (LPS) (19). Developing methods to understand neutrophil metabolism may provide insights into mechanisms underpinning disease and could offer a new therapeutic paradigm, improving outcomes for patients with acute and chronic disease.

Extracellular Flux (XF) analysis assesses real-time measurement of oxygen consumption rate (OCR) and proton efflux rate (PER) of cells. XF technology allows automated addition of different stimuli or inhibitors with flexibility over timing to visualise the effect of stimulation and inhibitory factors on cellular metabolism. XF technology requires low cell numbers relative to other forms of respirometry and allows for simultaneous measurement of PER and OCR as indicators for glycolysis and mitochondrial function, respectively. The streamlined workflow combined with user-friendly interface and analysis templates have seen a worldwide boom in the use of these instruments for probing cellular metabolism. In this manuscript we describe optimised protocols for using an XFe96 XF Analyser to probe (i) glycolysis in neutrophils under basal and stimulated conditions and (ii) phorbol 12-myristate 130-acetate (PMA)-induced oxidative burst. We highlight limitations of using XF technology to examine mitochondrial function in neutrophils. Finally, we provide an overview of how to analyze XF data and identify pitfalls of probing neutrophil metabolism with XF analysis.

## Materials, equipment and methods

### Study subjects

11 otherwise healthy volunteers aged 22-49 years old were recruited from staff members in the Queen Elizabeth Hospital Birmingham. Written informed consent was given by all participants in accordance with the University of Birmingham Research Ethics Committee (ERN 12-1185R2).

### Isolation of blood neutrophils

Neutrophils are notoriously challenging cells to work with due to their short lifespan and propensity for activation. The upmost care must be taken during cell isolation and assays should be commenced immediately following isolation to reduce risk of apoptosis.

Peripheral blood neutrophils were isolated from lithium-heparin anticoagulated (BD Bioscience, New Jersey, USA) whole venous blood as previously described (20). Briefly, whole blood was mixed 6:1 with 2% dextran (Merck Life Sciences, UK) (w/v in 0.154 M saline) for 30 minutes to allow erythrocyte sedimentation. Percoll (GE Healthcare, New York, USA) was made using 9:1 v/v Percoll to sterile saline (1.54 M) which was further diluted to 56% and 80% v/v solutions in 0.154 M saline. The buffy coat was layered on Percoll discontinuous gradients and centrifuged at 470 x *g* for 20 minutes with no brake or acceleration. Neutrophils were removed and washed in phosphate-buffered saline (Merck Life Sciences, UK) (PBS) (250 x *g*, 10 minutes, full brake and acceleration) before being resuspended at 4 x 10^6^/mL in phenol red and sodium bicarbonate free, filter sterilized Roswell Park Memorial Institute-1640 (RPMI) (Merck Life Sciences, UK, R8755), supplemented with 2mM L-glutamine, 1mM sodium pyruvate, 25mM glucose and 5mM 4-(2-hydroxyethyl)-1-piperazineethanesulfonic acid (HEPES) (Merck Life Sciences, UK), pH 7.4, which will be referred to as XF media. Neutrophil purity was >99% for all assays, except where neutrophil purity is detailed in the Figure legend.

### Extracellular flux assays

All assays were carried out using XFe96 V3 PS Cell Culture microplates (Agilent Technologies, California, USA) coated with 22.4 µg/mL Corning^®^ Cell-Tak™ (Corning Inc., New York, USA) solution and stored overnight at 4°C. On the day of the assay pre-coated microplates were bought to room temperature and seeded with 2 x 10^5^ cells/well (glycolysis and mitochondrial respiration) or 5 x 10^4^ cells/well (oxidative burst) in 50µL XF media. Wells A1, A12, H1 and H12 contained only 50 µL/well XF media to account for background correction wells. The XFe96 microplate was centrifuged at 200 x *g* for 1 minute with maximum acceleration but no brake at room temperature before incubation in a non-CO_2_, 37°C incubator for 25-30 minutes. Wells were observed under a light microscope to ensure cell adherence before gently adding 130 µL/well pre-warmed XF media, after addition of media the plate was incubated for a further 15 minutes in a non-CO_2_, 37°C incubator. 25 µL of each inhibitor/stimulant was added to injection ports A-C of the sensor cartridge (Agilent Technologies, California, USA) as follows, with stated concentrations referring to final well concentrations: *Glycolysis-* port A: 160 nM PMA (Merck Life Sciences, UK), port B: 2 µg/ml oligomycin (21) (Merck Life Sciences, UK), port C: 50 mM 2-DG (22) (Merck Life Sciences, UK). *Mitochondrial respi ration* - port A: 2 µg/ml oligomycin, port B: 3 µM BAM-15 (21) (Bio-techne, Minneapolis, USA) and port C: 2 µM rotenone/antimycin A (21) (Merck Life Sciences, UK). *Oxidative burst* - port A: XF media, port B: 2 µM rotenone/antimycin A and port C: 160 nM PMA. Or port A: 2 µM rotenone/antimycin A, port B: 10ng/ml tumour necrosis factor α (TNFα) (Merck Life Sciences, UK) or XF media, port C: compound of interest. *N*-Formyl methionyl-leucyl-phenylalanine (fMLP) (Merck Life Sciences, UK). Heat killed *Streptococcus pneumoniae* (SP) and non-typeable *Haemophillus influenzae* (NTHI) were donated by Dr Kylie Belchamber (23). Mitochondrial damage associated molecular patterns (mtDAMPS) were donated by Dr Jon Hazeldine (24). All compounds were prepared in XF media.

### Data analysis

OCR and extracellular acidification rate (ECAR data) were exported from WAVE to GraphPad Prism and Microsoft Excel for downstream analysis. Seahorse analytics (https://seahorseanalytics.agilent.com) was used to convert ECAR to PER using the buffer factor 2.8 as calculated by the manufacturer’s protocol (https://www.agilent.com/cs/library/usermanuals/public/usermanual-xf-buffer-factor-protocol-cell-analysis-S7888-10010en-agilent.pdf) for RPMI supplemented with 25 mM glucose. Glycolytic parameters were calculated as follows: Basal glycolysis - PER value prior to the first injection, PMA-induced glycolysis - maximum PER reached over the course of the assay, and glycolytic response to PMA – PMA-induced glycolysis minus basal glycolysis. Area under the curve analysis was performed on GraphPad Prism to generate oxidative burst parameters. Mitochondrial respiration parameters were calculated by importing OCR data into pre-formatted analysis templates in Microsoft Excel to calculate basal respiration, ATP-linked respiration, proton leak respiration, maximal respiration, and non-mitochondrial respiration. Mean and standard error of the mean (SEM) are presented for data. Paired t-test was performed for data 4A-B. Linear regression analysis was performed in GraphPad Prism for mitochondrial data in 8 A-D.

## Representative results

### Achieving a confluent monolater of neutrophils in XFe96 microwell plates

**Figure 1:**
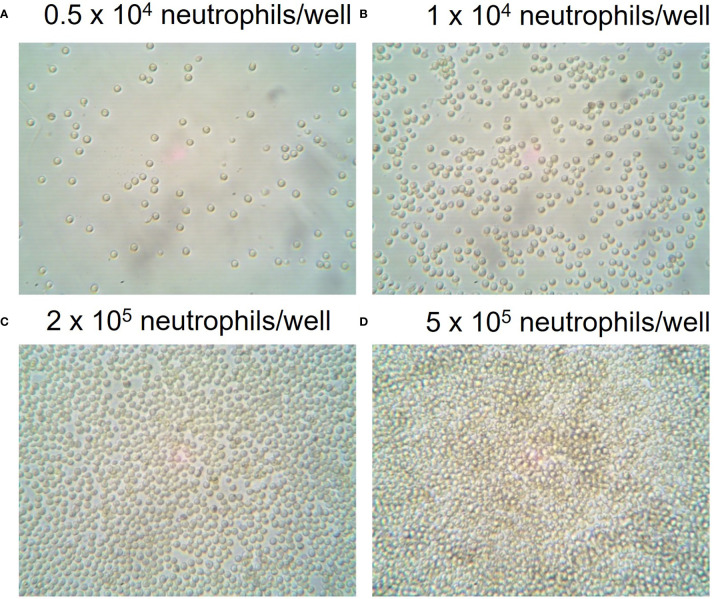
Seeding densities of neutrophils in XFe96 coated microplates. Human peripheral blood neutrophils were isolated from whole blood of donors before seeding into Cell-Tak™ coated XFe96 cell culture plates at **(A)** 1 x 10^4^, **(B)** 5 x 10^4^, **(C)** 2 x 10^5^ or **(D)** 5 x 10^5^/well. XFe96 cell culture microplates containing neutrophils were imaged under an inverted light microscope (45x magnification) and examined by eye for confluence percentage. Confluence assessments were combined with experimental data to achieve the optimal number of neutrophils per well for each test which achieved the correct confluence and consistent data outputs.

To establish optimal cell seeding concentrations, 1 x 10^4^ to 5 x 10^5^ neutrophils per well were seeded into Cell-Tak™ coated XFe96 microwell cell culture plates. Cell confluence was assessed by eye using an inverted optical light microscope. The manufacturer recommends cell confluence between 50-90% while avoiding cell clusters. **Figure 1** demonstrates 50-90% confluence for 5 x 10^4^ and 2 x 10^5^ neutrophils/well with a clear monolayer and no clustering of cells. Higher seeding densities resulted in cell clusters and disrupted the assessment of metabolism. Thus, a range of cell densities between 5 x 10^4^ and 2 x 10^5^ cells/well were examined for each XF assay to identify optimal cell densities.

### Oxidative burst

**Figure 2:**
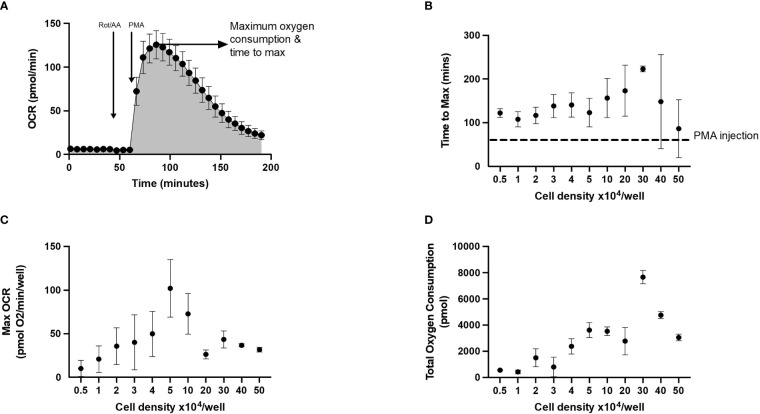
Representative trace and optimal cell seeding density to assess neutrophil oxidative burst using XF analysis. Neutrophils seeded at 0.5 – 5 x 10^4^/well in XFe96 cell culture microplates were sequentially injected with 1) XF media (injection 1), 2 µM rotenone/antimycin A (injection 2) and 160 nM phorbol 12-myristate 13-acetate (PMA) (injection 3). **(A)** is a representative oxygen consumption rate (OCR) trace (n = 1). Area under the curve analysis was performed, to calculate **(B)** time to maximum OCR, **(C)** maximum OCR reached and **(D)** total oxygen consumption over the duration of the test for each cell density (as shown in figure 2). Graphs B-D represent the mean ± standard error of the mean (SEM) from 3 distinct donors.

Oxygen consumption rate (OCR) measured by XF technology can be used to quantify oxidative burst response in mononuclear cells such as neutrophils. We highlight this in the representative oxidative burst trace in **Figure 2** using an acute injection of PMA to activate neutrophils seeded in XFe96 microplates. Consistent with an oxidative burst, PMA leads to a typical transient bell-shaped increase in oxygen consumption. Importantly, when rotenone plus antimycin A is added before PMA, mitochondrial complexes I and III are inhibited and the subsequent PMA-induced increase in OCR is independent of mitochondrial oxygen consumption. To quantify the oxidative burst, we assessed a) time to maximal oxygen uptake, b) maximum oxygen consumption as demonstrated by the arrow and c) total oxygen consumption after PMA injection (shaded area under the curve, **Figure 2A**). These metrics give a reliable quantitative comparison of oxidative burst between groups where both speed and magnitude of oxidative burst may be impacted.

#### Cell density and PMA titration

Given that both cell density and/or PMA concentration will affect the metrics used to quantify an oxidative burst using XF technology, we explored to what extent these factors effect neutrophil oxidative burst by assessing neutrophil cell density and PMA concentration. Initially, between 0.5 x 10^4^ and 5 x 10^5^ neutrophils/well were seeded onto XFe96 cell culture microplates as described in methods. Baseline OCR was measured over a period of 4 measurement cycles (2 min 30 sec mix, 30 sec wait, 3 min measure) prior to sequential injections of 2 µM rotenone plus antimycin A and 160 nM PMA to establish oxidative burst test parameters (**Figure 2A**). To quantify the oxidative burst time to maximum OCR (**Figure 2**) were calculated after injection of 160 nM PMA. Neutrophils seeded at 5 x 10^4^/well showed the greatest OCR of 102.12 pmol O_2_/min/well, while OCR decreased in cell densities greater than 5 x 10^4^/well (**Figure 2C**). Combining assessment of OCR from oxidative burst activity (**Figure 2**) with confluence assessments (**Figure 1**), we show that neutrophils seeded between 0.5 x 10^4^ and 5 x 10^4^ per well are optimal for assessment of oxidative burst.

**Figure 3:**
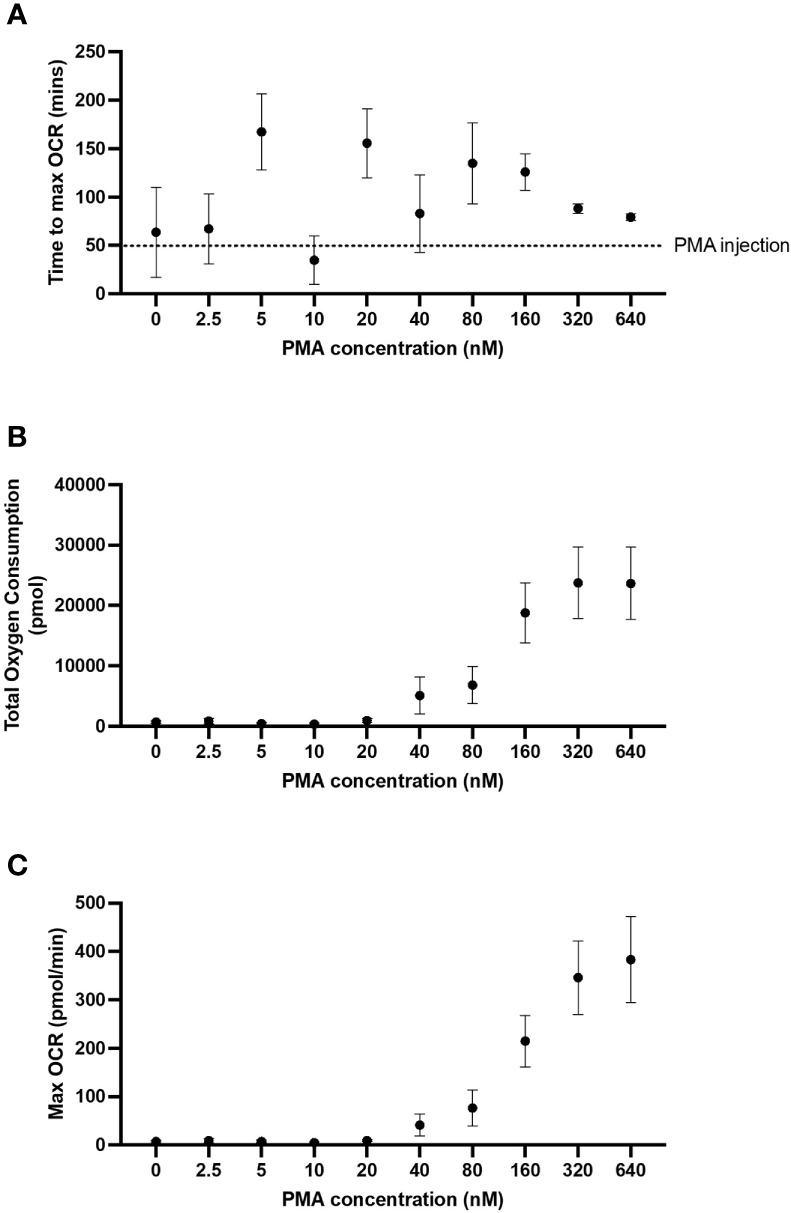
PMA-induced OCR from neutrophils. Human peripheral blood neutrophils were seeded at 5 x 10^4^ cells/well onto Cell-Tak™ coated XF XFe96 microplates. Oxidative burst was probed by sequential injection of media, 2 µM rotenone plus 2 µM antimycin A and phorbol 12-myristate 13-acetate (PMA) (640, 320, 160, 80, 40, 20, 10, 5 or 2.5 nM). Oxygen consumption rate (OCR) was monitored over the duration of the test, and results were exported to GraphPad Prism for downstream analysis. Time to max OCR **(A)**, maximum OCR **(B)** and total oxygen consumption **(C)** were calculated from area under the curve analysis. Graphs represent means ± standard error of the mean (SEM) from 5 distinct donors.

To establish the optimal dose of PMA required to induce an oxidative burst in 5 x 10^4^/well neutrophils, a dose response of PMA was performed including 640, 320, 160, 80, 40, 20, 10, 5 and 2.5 nM. PMA concentrations below 160 nM yielded inconsistent peaks of oxygen consumption (dotted line, **Figure 3A**). Both maximum OCR (**Figures 3B, C**) over the course of the assay were consistently increased from baseline at PMA concentrations above 160 nM. Based on our data in **Figure 3**, and in agreement with published literature (25, 26) we recommend using 160 nM PMA for assessing oxidative bursts from 5 x 10^4^/well neutrophils when using the XFe96 analyser. Additionally, 160 nM PMA allows for detection of both increases and decreases between groups, whereas higher concentrations result in the maximal oxidative burst response for 5 x 10^4^ neutrophils/well, so increases caused by disease states or pharmacological treatments would not be detected.

#### Assessment of oxidative burst induced by physiological stimuli

**Figure 4:**
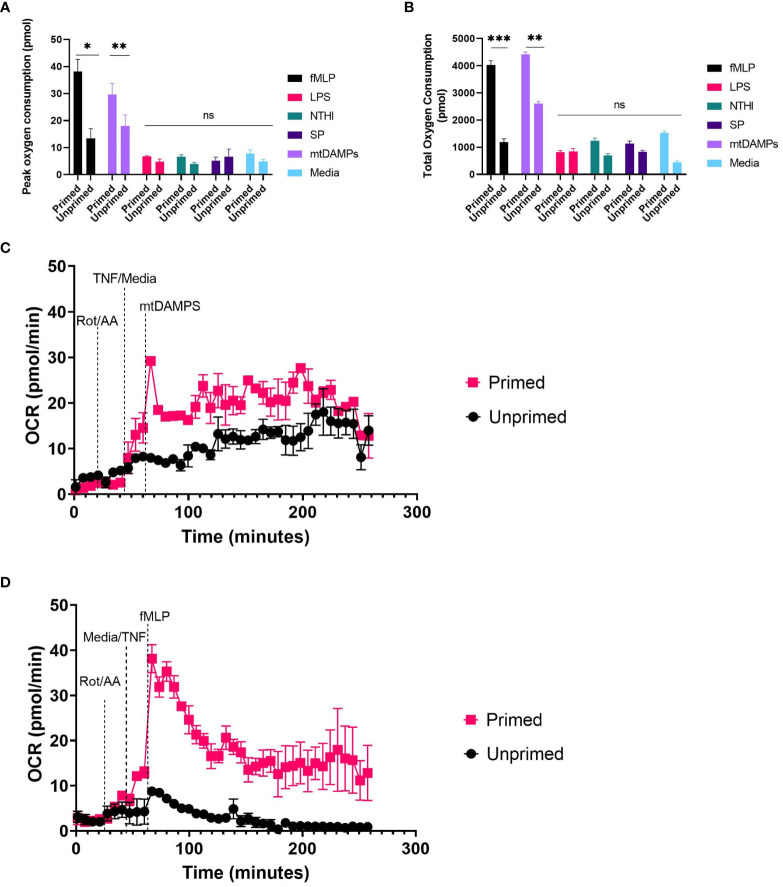
Induced OCR from activated neutrophils. Human peripheral blood neutrophils were seeded at 5 x 10^4^ cells/well onto Cell-Tak™ coated XF XFe96 microplates. Oxidative burst was probed by sequential injection of 2 µM rotenone plus 2 µM antimycin A, 10ng/ml TNFα (primed), or XF media (unprimed) followed by 10µM fMLP, or 10µm LPS, NTHI at 100 bacteria:1 neutrophil, SP at 100 bacteria: neutrophil or 100µg/ml mtDAMPS. Oxygen consumption rate (OCR) was monitored over the duration of the test, and results were exported to GraphPad Prism for downstream analysis. Peak OCR **(A)** and total oxygen consumption (CB were calculated from area under the curve analysis. **(A, B)** represent means ± standard error of the mean (SEM) from 3 distinct donors. **(C, D)** are representative traces from two distinct donors. Data for 100ng/ml PMA not shown. A) fMLP primed vs. unprimed P= 0.024, mtDAMPs p=0.0037, others non-significant. B) fMLP p=0.0001, mtDAMPs p=0.0036, others non-significant. fMLP, *N*-Formyl methionyl-leucyl-phenylalanine; LPS, lipopolysaccharide; NTHI, non typeable *Haemophillus influenzae; SP, Streptococcus pneumoniae*; mtDAMPS, Mitochondrial damage associated molecular patterns; OCR, oxygen consumption rate; TNFα, tumour necrosis factor. ns, p >0.05; *, p ≤ 0.05; **, P ≤ 0.01; ***, P ≤ 0.001.

Using neutrophils seeded at 5x10^5^ cells/well we explored the effect of physiological stimuli confirmed to generate reactive oxygen species by other methods (27–30). Experimental procedure was as described in methods, except injection strategy was A:2 µM rotenone plus antimycin A, B:10ng/ml TNFα or XF media, C: fMLP, LPS, heat killed bacteria or mtDAMPS at a range of concentrations. Unprimed neutrophils did not generate a detectable increase in oxygen consumption when challenged with heat killed *SP*, *NTHI* or LPS at a range of concentrations. fMLP at 0.01µM- 10µM generated a small and rapid peak in OCR (13.35-8.11 pmol O_2_/min/well) three minutes following injection (**Supplementary Figure 1**). mtDAMPs generated a peak oxygen consumption of 18.01 pmol O_2_/min/well 130 minutes following injection without priming (**Figure 4**).

Priming with TNFα results in significantly greater oxygen consumption when neutrophils are challenged with fMLP (p <0.001) and mtDAMPs (p= 0.002) compared to unprimed neutrophils. Peak oxygen consumption is rapid within three minutes of injection and overall oxygen consumption is lower than that induced by PMA. Peak oxygen consumption induced by fMLP was 38.12 pmol O_2_/min/well fMLP and 29.7 pmol O_2_/min/well by mtDAMPs (**Figure 4A**). Total oxygen consumption was greatest by mtDAMPs 4409 pmol compared to 4033pmol induced by 10µM fMLP. TNFα priming does not result in detectable oxygen consumption when neutrophils are challenged with heat killed bacteria or LPS compared to XF media alone (**Figure 4**). Treatment with TNFα alone resulted in a small increase in total oxygen consumption 1533pmol compared to 443pmol in unstimulated neutrophils (**Figure 4**). Therefore, we recommend use of priming agents prior to testing compounds other than PMA when investigating oxidative burst by XF analyser, and titrating cell density to achieve OCR well within the limits of detection.

### Glycolytic assessment

#### Extracellular acidification rate and proton efflux rate

XF analysis can also provide detailed information on cellular glycolytic flux. Until recently (31), ECAR was commonly used as an indicator of cellular glycolytic rate, given that at neutral pH conversion of glucose to lactate releases protons and acidifies the medium. However, the relationship between extracellular acidification and glycolytic rate can be confounded by other acidification mechanisms. Specifically, in cells that have active mitochondrial oxidative phosphorylation, CO_2_ generated in the tricarboxylic acid cycle can be spontaneously or enzymatically hydrated to carbonic acid (H_2_CO_3_), which dissociates to $\text{HC}{\text{O}}_{3}^{-}$ + H^+^ in media at physiological pH. Of significance, conversion of one glucose molecule to lactate yields 2 lactate + 2 H^+^, whereas complete oxidation of one glucose to CO_2_ yields 6 $\text{HC}{\text{O}}_{3}^{-}$ + 6 H^+^, therefore when extracellular acidification of glucose is oxidized to CO_2_, extracellular acidification is three times higher than conversion to lactate. Moreover, XF analysis of ECAR from changes in media pH does not consider the buffering capacity of the media, which often contain low amounts of HEPES or other buffering agents. It is therefore also important to correct for buffering capacity of the XF assay media. This correction converts ECAR into proton efflux rate (PER), which is preferable when interpreting glycolytic flux.

#### Stimulant/activator

**Figure 5:**
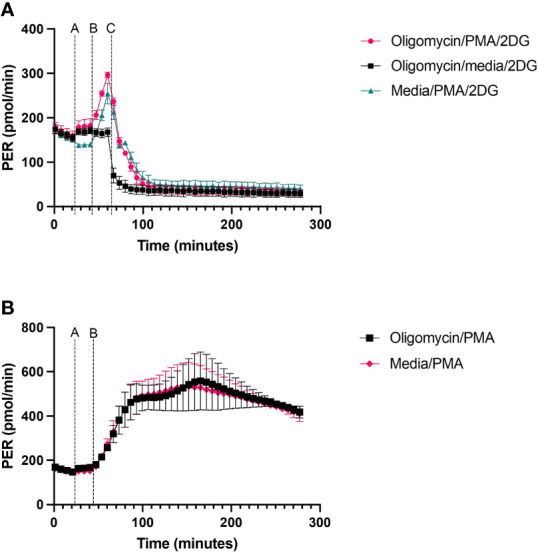
PER from neutrophils exposed to oligomycin or PMA. Human peripheral blood neutrophils were seeded at a density of 2 x 10^5^ cells/well onto a Cell-Tak™ coated XFe96 microplate. **(A)** 2 µM oligomycin or XF media was injected first, followed by sequential injections of 160nM PMA or XF media and finally 50 mM 2-Deoxy-D-Glucose (2-DG) at defined time points as shown on the figure. **(B)** 2 µM oligomycin or XF media was injected first, followed by 160nM PMA. Extracellular acidification rate (ECAR) was measured across the duration of the test. Following data export, ECAR readings were converted to proton efflux rate (PER) using Seahorse analytics by accounting for the buffer capacity of the media. Data was exported to GraphPad Prism and expressed as mean ± standard error of the mean (SEM) from 3 distinct donors.

Glycolytic rate assessed using XF technology is obtained in cells exposed to exogenous glucose and mitochondrial complex inhibitors such as oligomycin or rotenone plus antimycin A. Typically, treatment of cells with mitochondrial respiratory complex inhibitors such as oligomycin increases PER because of increased glycolytic flux to sustain cellular ATP supply to meet the energetic needs of the cell when mitochondrial ATP synthesis is abolished. However, in neutrophils, under basal conditions with surplus exogenous glucose, oligomycin has no effect on PER (**Figure 5**), suggesting a lack of mitochondrial contribution to ATP supply in blood derived neutrophils. To measure increased glycolytic rate in neutrophils a stimulation step is required to increase ATP hydrolysis rather than abolition of mitochondrial respiration. This is demonstrated in neutrophils treated ± PMA, whereby without PMA stimulation oligomycin has no impact on PER but when stimulated with PMA, PER increases more than 2-fold (**Figure 5A**). Oligomycin and PMA have no synergistic effect compared to PMA alone over 270 minutes (**Figure 5B**).

#### Cell density

**Figure 6:**
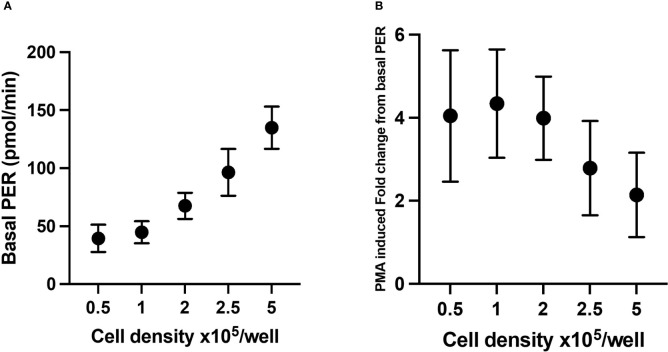
Glycolytic parameters from neutrophils seeded at different densities. Glycolytic rates of isolated neutrophils seeded at 0.5, 1.0, 2.0, 2.5 and 5 x 10^5^ cells/well were established from proton efflux rate (PER) data as described previously. Basal glycolysis **(A)** represents final baseline measurement of glycolysis prior to stimulation, **(B)** phorbol 12-myristate 13-acetate (PMA)-induced fold change from basal PER. Data represent means ± standard error of the mean (SEM) from 3 distinct donors.

To establish what cell seeding density is required to obtain consistent and reliable glycolytic rates from neutrophils stimulated with PMA, PER was assessed in XFe96 microplates containing 0.5 – 5 x 10^5^ isolated neutrophils per well. As indicated by increases in PER, basal glycolytic rate positively correlated with increased cell density (**Figure 6A**). However, PMA-induced fold change in PER was consistent only up to 2 x 10^5^ cells/well (**Figure 6B**). For this reason, we suggest that 2 x 10^5^ cells/well is an optimal cell density for assessing PER from neutrophils using the XFe96 analyser. This number of cells sits at the middle of the detectable baseline range and would allow for increases and decreases in glycolytic rate to be assessed under disease states. Importantly, 2 x 10^5^ cells/well also coincides with our confluence assessment (**Figure 1**).

### Mitochondrial respiration

#### Cell density

**Figure 7:**
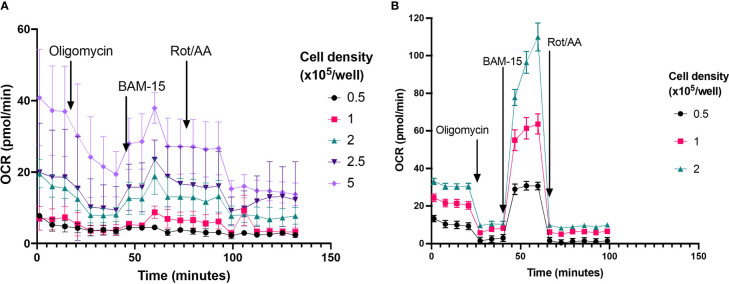
Probing OCR from neutrophils and PBMCs seeded at different cell densities. **(A)** Mitochondrial respiratory activity of isolated neutrophils seeded between 0.5-5 x 10^5^ cells/well were probed by sequential injection of 2 µM oligomycin (injection 1), 3 µM BAM-15 (injection 2) and 2 µM rotenone/antimycin A (Rot/AA) (injection 3), to establish rates of adenosine triphosphate (ATP)-coupled, maximal mitochondrial respiratory activity and non-mitochondrial oxygen consumption, respectively. Cell purity was assessed using H&E staining, mean neutrophil purity was 97% the contaminating cells were 2% monocytes and 1% lymphocytes. Data are means ± standard error of the mean (SEM) from 4 distinct donors **(B)** Mitochondrial respiration of isolated PBMCs seeded between 0.5-2x10^5^ cells/well were probed by sequential injection of 2 µM oligomycin (injection 1), 3 µM BAM-15 (injection 2) and 2 µM rotenone/antimycin A (Rot/AA) (injection 3), to establish rates of adenosine triphosphate (ATP)-coupled, maximal mitochondrial respiratory activity and non-mitochondrial oxygen consumption, respectively. Data are means ± standard error of the mean (SEM) 4 technical replicates.

To establish optimal neutrophil seeding density for assessment of mitochondrial respiration using XF analysis, a range of cell seeding densities were examined from 0.5 – 5 x 10^5^ cells/well. At cell concentrations less than 2 x 10^5^, basal OCR was less than 20 pmol O_2_/min/well (**Figure 7**), below the recommended threshold for OCR measured with the XFe96 analyser. Only at 2.5 and 5 x 10^5^ cells/well was OCR detectable above the 20 pmol O_2_/min/well threshold (**Figure 7** –Light and dark purple traces). Of interest, the typical OCR curve for assessing mitochondrial function with XF technology (32) is not apparent at lower cell seeding densities (**Figure 7**). Notably, OCR from 2.5 and 5 x 10^5^ cells/well do show sensitivity to the mitochondrial compounds oligomycin, BAM-15 and rotenone plus antimycin A, however there is considerable variability at such high cell seeding densities (**Figure 7**). Together with confluence images (**Figure 1**), at appropriate cell seeding densities (< 2 x 10^5^ cells/well), OCR is below the limit of detection for the XFe96 analyser and thus OCR obtained from neutrophils at these densities should be treated with caution when inferring mitochondrial respiratory activity.

#### Cell purity

Given that lower cell seeding densities (<2x10^5^ cells/well) make it difficult to obtain reliable OCR (**Figure 7**), it could be argued that increasing cell seeding would be beneficial for probing mitochondrial energy metabolism in neutrophils. That said, OCR measured during a mitochondrial stress test with higher neutrophil densities could be related to contamination from other mononuclear cells, which have higher mitochondrial activity. Typically, density gradient isolations are >97% pure, but contamination with other mononuclear cells such as monocytes or lymphocytes is a common issue when working with neutrophils.

**Figure 8:**
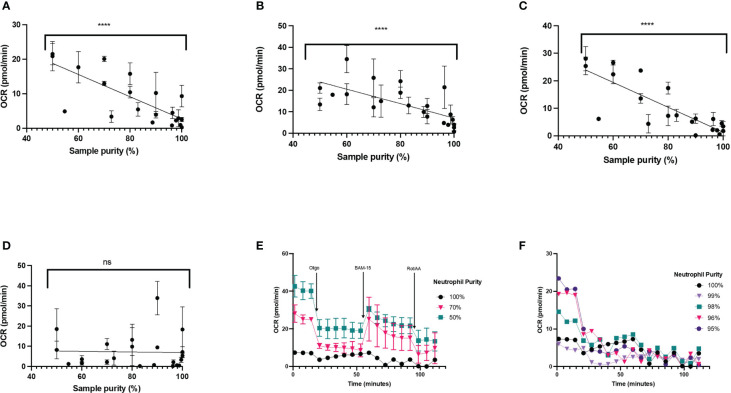
Mitochondrial respiration in varying neutrophil purities. Pure neutrophil isolations were contaminated with up to 50% peripheral blood mononuclear cells (PBMCs) harvested from the same donor and cells seeded at 2 x 10^5^/well. Mitochondrial respiration was probed for in neutrophils by sequential injection of 2 µM oligomycin, 3 µM BAM-15 and 2 µM rotenone plus 2 µM antimycin A (Rot/AA). **(A)** adenosine triphosphate (ATP)-coupled mitochondrial respiration, **(B)** maximal mitochondrial respiration, **(C)** basal mitochondrial respiration, **(D)** non-mitochondrial oxygen consumption, **(E)** mean oxygen consumption rate (OCR) ± standard error of the mean (SEM) over the duration of the assay, representing 100%, 70% or 50% neutrophil purities from 2 distinct donors and **(F)** mean oxygen consumption rate (OCR) ± standard error of the mean (SEM) over the duration of the assay, representing 100%, 99%, 98%, 96% and 95% neutrophil purities from a single donor. Data in **(A-D)** consists of three unique donors, for each donor the cell preparation was intentionally contaminated to achieve 22 samples with varying neutrophil purity between 100% neutrophils and 50% neutrophils. Data points shown are mean ± SEM for four technical replicates for each donor. ns, not significant; ****, p-value <0.0001.

To test this, we probed OCR in neutrophils contaminated with peripheral blood mononuclear cells (PBMCs) seeded at 2 x 10^5^ cells/well. This was achieved by mixing neutrophils with defined concentrations of PBMCs to yield cell populations containing either 100%, 70% or 50% neutrophils. OCR were significantly higher in cell populations that contain PBMCs (**Figures 8E, F**). In 100% pure neutrophil isolations, basal OCR was 4.5 (SEM 2.0) pmol O_2_/min/well compared to 26.72 (SEM 2.6) pmol O_2_/min/well at 50% purity (**Figures 8E, F**). Basal OCR appeared to be dose-dependently higher than pure neutrophil populations with increasing proportion of PBMCs (18-fold higher for 30% contamination and 26-fold higher for 50% contamination, compared to pure neutrophils) (**Figure 8E**). Also, the sensitivity of OCR to mitochondrial compounds is much greater from cell suspensions containing PBMCs (**Figure 8E**). OCR traces from 100% pure neutrophils fall below the 20 pmol O_2_/min/well threshold for the XFe96 analyser for the entirety of the mitochondrial assay, as shown in **Figure 8E**. We also show that basal OCR is different in neutrophil isolations between 95-100% purity (**Figure 8F**). When corrected for non-mitochondrial respiration (calculated as OCR after rotenone and antimycin A injection), parameters of mitochondrial respiration including basal mitochondrial respiration (**Figure 8C**), maximal respiration (**Figure 8B**) and ATP production (**Figure 8A**) correlate negatively with neutrophil purity (P < 0.001). It is also worth noting that non-mitochondrial oxygen uptake did not significantly change (P = 0.8252) with increasing PBMC concentration (**Figure 8D**).

## Discussion

Neutrophils are highly glycolytic cells (12); inhibition of glycolysis significantly impacts neutrophil effector functions (13). Neutrophils utilise glycogen stores to synthesise ATP in the absence of oxygen and are capable of gluconeogenesis to increase glycolytic capacity (11). The use of glycolytic inhibitors abolishes neutrophil ATP levels, while they remain unchanged after inhibition of mitochondrial respiratory complexes (33).

Activated neutrophils rapidly consume oxygen as reduced nicotinamide adenine dinucleotide phosphate (NADPH) oxidase forms superoxide anions, reactive oxygen species and hydrogen peroxide, a process commonly referred to as oxidative burst. PMA is a protein kinase C activator and a robust activator of neutrophils, albeit non-physiological (26). In cell seeding densities beyond 5 x 10^4^ neutrophils/well, we saw a reduction in the size of the oxidative burst response. Typically, it would be anticipated that greater cell seeding densities would produce a larger oxidative burst response, however, due to cell overcrowding and/or reduced PMA stimulation relative to number of cells, this was not the case. We demonstrated that 5 x 10^4^ neutrophils/well combined with an injection strategy incorporating 2 µM rotenone plus 2 µM antimycin A followed by 160 nM PMA, produces a substantial and consistent oxidative burst response, without maximising the neutrophil reactive oxygen species (ROS) response. This allows for detection of both elevated and suppressed oxidative burst responses caused by disease states or exogenous treatments. We show that PMA concentrations lower than 160 nM do not produce a consistent peak following PMA injection, and therefore area under the curve analysis is unable to quantify a reliable oxidative burst response. Data in **Figures 3**, **4** demonstrate that when assessing oxidative burst in neutrophils a multi-parameter approach should be taken as using a single parameter may lead to false assumptions as seen in the variability of time to max oxygen consumption (2B and 3A) which is due to detection of very small peaks at extremes of cell density and or PMA concentration, and are not likely to be a true difference in time to maximum oxygen consumption. PMA is a potent and non-physiological activator of neutrophils; therefore, we explored the utility of other stimuli in the oxidative burst assay. We demonstrated that priming with TNFα enhances detection of the increased oxygen consumption induced by fMLP and mtDAMPs. In unprimed cells there was a small increase in oxygen consumption following fMLP/mtDAMPs but this was well below the recommended limits of detection for OCR. Heat killed *SP*, NT*HI*, or LPS did not induce a detectable increase in oxygen consumption with or without priming. Heat killed bacteria and LPS have been used by others to induce oxidative burst, although with different methods (27, 28).

More commonly used methods to measure oxidative burst detect generation of ROS by a range of fluorescent, chemiluminescent probes rather than detecting environmental oxygen consumption (34). While these methods can be highly specific this can also be considered a weakness if assay conditions are not carefully controlled. Different probes have varying selectivity for different species of ROS and vary in their ability to detect intra and extracellular ROS (34), this is frequently an issue in commercially available kits where the selectivity is not always clearly documented. In addition, chemiluminescent probes such as luminol and lucigenin are prone to errors related to redox recycling as the probes can generate radicals that produce superoxide (35). Using the XF analyser to assess oxidative burst allows for measurement of the total oxygen uptake from the environment which may better represent total ROS generation, especially when mitochondrial uptake of oxygen is abolished. This assay typically uses half the cell number of chemiluminescent assays and enables simultaneous indication of glycolysis through PER data.

Neutrophils rely on glycolysis for synthesis of ATP. In the traditional glycolytic rate test (22) used in other cell types, injection of oligomycin (ATP synthase inhibitor) or rotenone and antimycin A pushes glycolysis to its capacity, as cells are unable to produce ATP *via* mitochondrial respiration and therefore upregulate glycolysis to meet the energetic demands of the cell. When testing this assay on human neutrophils, we observed no change in glycolysis after oligomycin injection. A recent publication has highlighted that by inhibiting mitochondrial respiration using mitochondrial respiratory complex inhibitors in resting state cells, cells only shift to glycolysis as a compensatory mechanism for basal ATP demand requirements, thus these agents may not actually shift cells to maximum glycolytic capacity as previously thought (36). This confirms our findings, highlighting the need for a stimulant to increase ATP demand. Here we used 160 nM PMA, to increase glycolytic activity in neutrophils and assess downstream glycolytic parameters. Seeding more than 2 x 10^5^ neutrophils per well to probe glycolytic activity resulted in inconsistent and lower glycolytic responses. Similarly, to the oxidative burst test, this is likely due to overcrowding of cells in the bottom of the well, which compromises the monolayer required to run these assays.

The manufacturer states a limit of detection of 20 pmol O_2_/min/well for basal OCR from cells assessed with the XFe96 and XFp analyzers. OCR below this threshold cannot be interpreted reliably and should be used with caution to infer mitochondrial function. Here, neutrophil mitochondrial metabolism is too low to detect using current XF analyzers – OCR are below the limit of detection and do not change in response to mitochondrial stressors. Moreover, this was not overcome by increasing cell seeding density. In fact, our data showed that increases in cell seeding density yields amplified the low levels of contamination by mononuclear cells which appear to skew mitochondrial respiration tests using XF analysis. These data highlight the importance of using highly pure neutrophil preparations when performing metabolic assays and suggest caution to be taken when inferring mitochondrial respiration from OCR of neutrophil preparations.

Of interest, in two recent studies, a typical mitochondrial OCR trace using XFe96 technology was reported in neutrophils seeded between 0.5-1.5 x10^5^ cells/well with isolation purities >95% (19, 37). At similar cell seeding densities, in our study we were unable to achieve equivalent traces. Such responses were only apparent with much less pure (<70%) neutrophil preparations. We show that basal OCR are higher in preparations with greater PBMC contamination at levels typically deemed acceptable for neutrophil assays (7E), however these data should be interpreted with caution given the lower limit of detection for XF analyzers. Given that PBMCs have higher rates of mitochondrial respiration than neutrophils, our data demonstrated that contamination with PBMCs has significant implications when assessing mitochondrial respiratory parameters from isolated neutrophils. Notably, in both papers, basal OCR are below the optimal minimum detection threshold for the XFe96 analyser. Additionally, the results as presented are problematic. For example, Porter et al. show higher OCR in neutrophils after rotenone and antimycin A injection than basal OCR, suggesting a negative basal rate of mitochondrial respiration (38). Unless non-mitochondrial oxygen consumption is stimulated in such experiments, for example by increased ROS, such finding is not theoretically possible. Nonetheless, if increased ROS are responsible for such increases in OCR this needs to be corrected to accurately calculate parameters of mitochondrial function.

Consistent with our findings, Chacko et al. show that PER is not responsive to oligomycin in neutrophils (39). Moreover, neutrophil OCR is not sensitive to mitochondrial compounds, corroborating our findings and agreeing with the central dogma for a minimal role of mitochondrial oxidative phosphorylation in neutrophil energy generation. Our data, combined with existing evidence, suggests that mitochondrial ATP synthesis is below the limit of detection for XF analyzers, and thus play an insignificant role in supplying ATP to meet basal energetic demands in mature neutrophils. Mitochondrial ATP synthesis does appear to be required for messenger purposes (13, 40). Further, mitochondrial respiration appears to be important in supporting oxidative burst in the absence of glucose by providing NADPH oxidase (41).

XF analyzers account for the pH and oxygen content in background wells, which contain no cells. This enables background correction of the values to ensure readouts represent actual metabolism rather than changes in ambient O_2_ concentration or pH fluctuations affecting media parameters. For adherent cell cultures, normalisation is required to account for well-well cell density differences across the plate. When using neutrophils, cells are plated from pre-diluted homogenous cell suspensions at defined cell seeding densities per well and therefore normalisation to cell number, protein content or DNA will have modest effects on OCR or ECAR. This is, of course, if even cell seeding across the plate is obtained by the experimenter. Reporting ECAR values (traces or extrapolated values) are no longer accepted as representative for glycolysis. Media often has a large buffering capacity, which by its nature can mask pH changes, and subsequent acidification rates. Therefore, by calculating and applying a buffering factor for each specific media to account for the buffering capacity, ECAR values can be converted to PER, a more representative readout for glycolytic tests (when corrected for any mitochondrial-linked PER).

XF analyzers allow for high throughput data and multiple metabolic tests to be performed on a single assay plate, with a minimal demand for cell numbers. The dynamics of oxygen consumption and change in pH over the duration of the assay allows for the depiction of cellular metabolism and metabolic responses over time. XF analyzers accurately predict lactate production compared to direct lactate measurement (42). Other methods to assess glycolysis include direct measurement of glucose and or lactate in assay media, use of glucose analogs, measurement of rate limiting glycolytic enzymes, glucose tracing and metabolite quantification. Each of these has strengths and limitations, we recommend a combined approach to interrogation of metabolism for robust data. These are discussed in detail in (31). Alternative respirometers such as the Oroboros O2k analyser require 2mL of solution, with a recommended 2-4 million cells (based on HL-60 data) per chamber (43, 44), more than 40x the requirement for a well of a XFe96 cell microplate. XF technology also incorporates a pH probe to allow the determination of the glycolytic pathway alongside oxygen consumption simultaneously, without the need for additional modules. As mentioned previously, neutrophils are notoriously easy to activate by mechanical, chemical, and biological stressors. Respirometers such as Oroboros allow for inhibitor/stimulant injections but operate using continuous stirring motions, which may activate neutrophils, skewing metabolic readouts. Adherent cells lend themselves naturally to XF assays, as they can be cultured in a monolayer directly in the cell plate. By incorporating a molecular adhesion step, cells in suspension can be manipulated into a monolayer to perform metabolic assays without exposing them to rigorous mixing. It has been demonstrated previously that plate adhesion using Cell-Tak™ does not affect neutrophil ROS production (37).

XF analyzers are highly sensitive; however, care must be taken in ensuring an evenly distributed monolayer of cells is formed. If cells are not distributed directly under the probe, or if they begin to detach from the base of the well due to disruptions, probes can lack fine discrimination. Further, introduction of pharmaceutical treatments or other stimulants/inhibitors requires further assay validation to ensure they do not interact with the probes themselves, which are sensitive to pH. Despite these limitations, we have demonstrated how XF technology can be harnessed for neutrophil bioenergetic analysis. XF analyzers are costly to run compared to other respirometers, however the high throughput and automated nature of the system allows for efficient data collection.

In conclusion, we have presented optimised protocols for the assessment of neutrophil glycolysis and oxidative burst using XF technology. The metabolic tests outlined here can be applied to any project context by incorporating pharmacological treatments, disease state neutrophils or neutrophils from different developmental stages. We demonstrate robust methodology to achieve neutrophil monolayers to perform metabolic assays for assessing glycolytic rate and oxidative burst. We highlight the challenges of these assays and mitigating strategies when assessing mitochondrial respiration. Overall, XF technology represents a powerful platform with a user-friendly interface and data analysis templates which has the potential to revolutionise our understanding of neutrophil metabolism. The protocols described in our manuscript provide a template to enhance discovery and ensure a standardised approach to neutrophil measurements using XF analysis.

## Data availability statement

The raw data supporting the conclusions of this article will be made available by the authors, without undue reservation.

## Ethics statement

The studies involving human participants were reviewed and approved by University of Birmingham Research Ethics Committee (ERN 12-1185R2). The patients/participants provided their written informed consent to participate in this study.

## Author contributions

AJ and FG performed all experiments. JB, AS, AJ and FG designed the study with critical review by DT, ES and CM. Data analysis was undertaken by AJ, FG, and JB. All authors contributed to the article and approved the submitted version.
