# Supplementary material for: Real-time assessment of neutrophil metabolism and oxidative burst using extracellular flux analysis
Source: Front Immunol. 2023 Apr 25;14:1083072. doi: 10.3389/fimmu.2023.1083072 (PMC10166867; doi:10.3389/fimmu.2023.1083072)
Supplement: Supplementary file 1 [file DataSheet_1.docx]

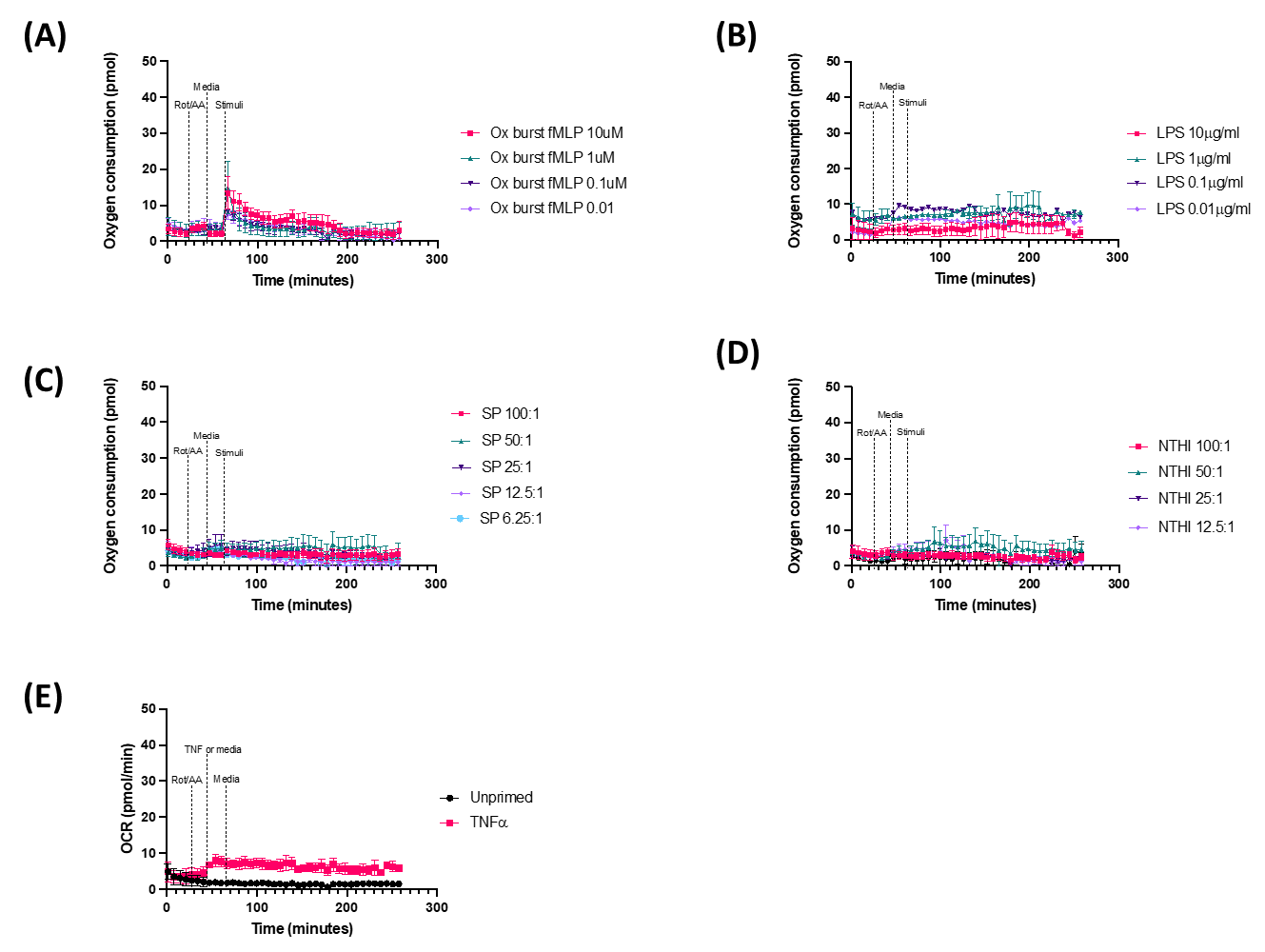
**Supplementary Figure 1: Induced OCR from activated neutrophils using a range of compounds.** Human peripheral blood neutrophils were seeded at 5 x 10^4^ cells/well onto Cell-Tak™ coated XF XFe96 microplates. Oxidative burst was probed by sequential injection of 2 µM rotenone plus 2 µM antimycin A, XF media (unprimed) followed by fMLP, or LPS, NTHI, SP at a range of concentrations. Oxygen consumption rate (OCR) was monitored over the duration of the test, and results were exported to GraphPad Prism for downstream analysis. A: fMLP, b: LPS, C: SP, D: NTHI, E: treatment with 10ng/ml TNFα with no subsequent stimuli. Data shown are from three distinct donors.

fMLP: *N*-Formylmethionyl-leucyl-phenylalanine, LPS: lipopolysaccharide, NTHI: non typeable *Haemophillus influenzae, SP: Streptococcus pneumoniae* , mtDAMPS:Mitochondrial damage associated molecular patterns, OCR:oxygen consumption rate, TNFα: tumour necrosis factor.
